# Plant Viruses as Nanoparticle-Based Vaccines and Adjuvants

**DOI:** 10.3390/vaccines3030620

**Published:** 2015-08-05

**Authors:** Marie-Ève Lebel, Karine Chartrand, Denis Leclerc, Alain Lamarre

**Affiliations:** 1 Immunovirology laboratory, Institut national de la recherche scientifique, Institut Armand-Frappier, 531 Boulevard des Prairies, Laval, QC H1V 1B7, Canada; E-Mails: Marie-Eve.Lebel@iaf.inrs.ca (M.-E.L.); Karine.Chartrand@iaf.inrs.ca (K.C.); 2 Department of Microbiology, Infectiology and Immunology, Infectious Disease Research Centre, Laval University, Quebec City, QC G1V 4G2, Canada; E-Mail: Denis.Leclerc@crchudequebec.ulaval.ca

**Keywords:** recombinant plant virus, vaccine, adjuvant, immune response, production methods

## Abstract

Vaccines are considered one of the greatest medical achievements in the battle against infectious diseases. However, the intractability of various diseases such as hepatitis C, HIV/AIDS, malaria, tuberculosis, and cancer poses persistent hurdles given that traditional vaccine-development methods have proven to be ineffective; as such, these challenges have driven the emergence of novel vaccine design approaches. In this regard, much effort has been put into the development of new safe adjuvants and vaccine platforms. Of particular interest, the utilization of plant virus-like nanoparticles and recombinant plant viruses has gained increasing significance as an effective tool in the development of novel vaccines against infectious diseases and cancer. The present review summarizes recent advances in the use of plant viruses as nanoparticle-based vaccines and adjuvants and their mechanism of action. Harnessing plant-virus immunogenic properties will enable the design of novel, safe, and efficacious prophylactic and therapeutic vaccines against disease.

## 1. Introduction

Although vaccines have allowed great achievements such as a significant reduction in incidence, mortality, and morbidity related to infectious diseases, the field of vaccinology has faced several challenges in recent years. Indeed, development of new vaccines has proven difficult especially against chronic infections or cancer. To overcome these obstacles, large research efforts are being devoted to better understand the key elements required to generate protective immunity. For example, while the majority of available vaccines mainly induce the generation of antibodies that neutralize targeted pathogens, it is now recognized that the cellular immune response is often necessary to protect against many infections. A balanced and complete immune response involving both humoral immunity, with high-affinity neutralizing antibodies limiting pathogen transmission and cellular immunity, with cytotoxic CD8^+^ T cells killing infected cells, is in fact required to control malaria, hepatitis C, and HIV/AIDS, just to list a few [1,2,3,4,5], highlighting the need for the development of new vaccine design approaches. Moreover, antigens and epitopes from several pathogens targeted by protective immune responses have recently been identified [6,7,8,9]. This has led to the design of safer and more specific recombinant vaccines, although they are often less immunogenic than more traditional vaccines based on live/attenuated pathogens. Therefore, the choice of vaccine formulations and the addition of suitable adjuvants will likely be required to achieve protective immunity with these novel vaccines. It is generally accepted that the best way to generate vaccines capable of generating a broad immune response with high levels of antibodies and cytotoxic T lymphocytes is to mimic a pathogenic infection while remaining as safe as possible. In this regard, virus-like nanoparticles (VLPs) have been increasingly studied in recent years with the aim of developing new effective vaccines.

## 2. Virus-Like Nanoparticles

VLPs mimic viruses by bearing similar protein composition while being non-infectious. In fact, most VLPs do not contain any viral genetic material while others contain nucleic acids that do not allow virus replication in mammals. For now, there are three vaccines composed of VLPs used in humans: the hepatitis B vaccine, the human papilloma vaccine, and the recently approved vaccine against hepatitis E in China [10]. These generate robust humoral immune responses but still require the addition of an adjuvant to be fully effective. Other VLPs are currently in clinical trials [11,12,13] and many more are in development [14,15,16]. The increase in VLP use in the development of novel vaccines is related to their numerous qualities that meet new medical needs. For example, their highly ordered and repetitive structures facilitate recognition by the immune system and induce B-cell activation through B-cell receptor (BCR) cross-linking [17]. The size of VLPs is generally between 20 and 300 nm, which is ideal to be effectively recognized by dendritic cells (DCs) or other antigen-presenting cells (APC) [18,19,20,21]. In addition, VLPs frequently display various Pathogen Associated Molecular Patterns (PAMPs) that are recognized by Pattern Recognition Receptors (PRRs), leading to the activation of the innate immune system and cytokine production such as type I interferon (IFN-I); notably, these cytokines were reported to increase the cross-presentation capacity of DCs and are beneficial towards cell-mediated, T-lymphocyte immune responses [22,23,24]. IFN-αβ can also increase the humoral response, induce isotype switching, and enhance the generation of T follicular helper cells [25,26]. Furthermore, many studies have shown the capacity of VLPs to induce a cellular immune response, therefore permitting the development of prophylactic and therapeutic vaccines targeting CD8^+^ cytotoxic T lymphocyte (CTL) responses [27,28,29,30]. This strongly correlates with APC cross-presentation of antigens fused to VLPs to CD8^+^ T cells through a Tap- and proteasome-independent pathway [31,32,33]. This mechanism has also been shown to generate tumor-specific CD8^+^ T-cell responses that efficiently slow down tumor growth and increase mice survival in various models [34,35,36]. Importantly, non-infectious VLPs are safer than attenuated or inactivated viruses and could therefore potentially be administered to immunocompromised individuals. Finally, the use of VLPs could be advantageous for developing vaccines against viruses that are impossible or difficult to grow in culture such as the human norovirus, for example [37]. Altogether, these properties contribute to the effectiveness and usefulness of VLP vaccines.

## 3. Recombinant Plant Virus Particles (rPVPs)

Recombinant plant virus particles are increasingly studied as candidate vaccines. They can be classified as a subtype of VLP since they are viral particles that are non-infectious in mammals. However, most rPVPs retain their replication potential in plants, posing additional safety and environmental challenges. Nonetheless, rPVPs possess all of the advantages of VLPs and thus would allow the development of effective vaccines. Many plant viruses such as the tobacco mosaic virus (TMV), cowpea mosaic virus (CPMV), potato virus X (PVX), alfalfa mosaic virus (AlMV), and papaya mosaic virus (PapMV) (see Table 1 for more information) are currently used for the development of new vaccines. Typically, plant viruses are simple, rod shaped, or bearing icosahedral symmetry; they are composed of one or two repeated coat protein (CP) subunits and bear an RNA genome. These viruses are relatively easy to engineer, produced at low cost, and very stable, enabling storage at room temperature, which is desirable for vaccination in developing countries. CP modification of plant viruses allows dense expression of fused antigens, thereby contributing to the development of an effective immune response. Finally, while other virus platforms, such as adenovirus, suffer from platform-specific antibody development [38,39], rPVPs seem to maintain their immunogenic properties in the presence of specific antibodies [40,41]. In the following paragraphs, we will discuss the different methods of production of rPVPs for vaccination, the diverse approaches for antigen expression on their surface, and achievements made with rPVPs.

**Table 1:** Recombinant plant virus particles used in vaccine development.

Virus	Shape	Parameters Tested	Element of Response	References
CPMV	Icosahedral	Humoral response	Binding or neutralizing antibodies	[42,43,44,45,46,47,48,49,50,51,52,53,54]
			Protection against challenge	[42,43,45,47]
		Cellular response	IFN-γ production	[50,51]
		Immunomodulation	APC activation	[55]
PVX	Rod	Humoral response	Binding or neutralizing antibodies	[56,57,58,59,60]
			Protection against challenge	[60]
		Cellular response	CD8^+^ T cells activation	[57,61]
			IFN-γ production	[57,61]
			Protection against challenge	[57,61]
		Immunomodulation	APC activation	[56,60]
			Cytokine production	[60]
TMV	Rod	Humoral response	Binding or neutralizing antibodies	[41,61,62,63]
			Protection against challenge	[41,63,64,65,66]
		Cellular response	CD8^+^ T cells activation	[40,67,68]
			IFN-γ production	[67]
			Protection against challenge	[67]
		Immunomodulation	APC activation	[40,67]
CMV	Icosahedral	Humoral response	Binding or neutralizing antibodies	[69]
		Cellular response	CD8^+^ T cells activation	[69]
			IFN-γ production	[69,70]
AlMV	Icosahedral	Humoral response	Binding or neutralizing antibodies	[71,72]
		Cellular response	IFN-γ production	[72]
PapMV	Rod	Humoral response	Binding or neutralizing antibodies	[73,74,75]
		Cellular response	CD8^+^ T cells activation	[33,76,77]
			IFN-γ production	[33,74,76,77]
			Protection against challenge	[77]
		Immunomodulation	APC activation	[78]
			Cytokine production	[78]
			Protection against challenge	[78]
		Adjuvant	APC activation	[79,80]
			Cytokine production	[79,80]
			Vaccine jointly administered	PapMV-M2e [73], BMDC-OVA [81], Outer Membrane Protein C [80], TIV [81],
			Protection against challenge	[79,80,81]
BaMV	Rod	Humoral response	Binding or neutralizing antibodies	[82,83]
			Protection against challenge	[82,83]
		Cellular response	IFN-γ production	[82]
			Protection against challenge	[82]
TBSV	Icosahedral	Humoral response	Binding or neutralizing antibodies	[84]
Plum pox potyvirus	Rod	Humoral response	Binding or neutralizing antibodies	[85,86]

CMV: cucumber mosaic virus; BaMV: bamboo mosaic virus; TBSV: tomato bushy stunt virus.

### 3.1. Production Methods

rPVP production can be achieved by several methods. Production of rPVPs using plants, such as *Nicotiana benthamiana*, *Nicotiana tabacum*, or cowpea plants, is the most widely used and can be divided into two different strategies, both starting with the production of the desired cDNA. The cDNA can then be transcribed *in vitro* and used to inoculate plants depositing the RNA transcript on abraded leaves to induce a systemic infection [64]. Another option is to transform *Agrobacterium tumefaciens* with a plasmid containing the cDNA coding for the modified viral genome and then agroinfiltrate plants to induce transient expression and generate recombinant viruses [87]. A week or two later, recombinant viruses are then purified by different techniques [62,66,71]. Remarkably, due to the possibility of CMV production in edible vegetables, e.g., celery, lettuce, cucumber, tomato, carrot, pepper, and banana [88], plant production also advances the use of oral delivery of vaccine via ingestible plants [89,90]. This strategy would therefore reduce costs related to virus purification and eliminate the need for sterile needles and trained medical staff for vaccine administration. rPVP manufacturing *in planta* also allows for large-scale production devoid of contamination risks from human pathogens. However, a weakness of oral administration of rPVPs is related to the difficulty of controlling the amount of antigen taken by the patient and the potential development of tolerance to the antigen. In addition, genetic modifications that induce foreign antigen expression on plant virus proteins can sometimes affect viral replication, thus reducing production efficiency [88]. A new method based on transgenic plant cell suspension cultures was recently developed. This process, based on the culture of calli derived from transgenic plant lines expressing viral cDNA, allows for continuous production of large amounts of rPVPs with high reproducibility [91]. More conventional techniques are also used to produce rPVPs such as bacteria [78,92], yeast [93,94], and insect cell cultures [95,96]. Plants, bacteria, and yeast are all simple and low cost manufacturing methods. However, bacteria and yeast sometimes produce insoluble proteins, thus restricting particle self-assembly [94,97]. The less practical and more costly baculovirus expression system seems to avoid these problems [94,95,96]. When capsid protein production does not induce particle formation, it is also possible to perform the assembly process *in vitro*, with or without the addition of nucleic acids [78,97,98,99]. Although this process is more laborious, it allows for better control of the RNA present inside the particle since diverse and uncontrolled sources of RNA can be incorporated in self-assembled viral particles *in vivo* [93,94,96]. In addition, *in vitro* assembly allows for the packing of specific RNA transcripts in recombinant plant virus capsid proteins that will further induce *in vivo* gene expression [100]. Atabekov and colleagues generated spherical nanoparticles using thermal denaturation of the TMV CP protein [101]. These particles are devoid of RNA and can bind different proteins or peptides, making it a universal and immunogenic particle platform [102]. Therefore, these different manufacturing processes generate rPVPs that either contain no RNA, host RNA, viral RNA, or inactive or replicative synthetic RNA. Plant virus particles are most probably safe enough for administration in humans, but many are still infectious in plants. Therefore, inactivation methods based on chemical treatment or UV irradiation, for example, were developed to ensure that rPVPs are innocuous [43,103,104]. Finally, methods using eukaryotic cells have the advantage of allowing post-transcriptional modifications ensuring that the rPVP is more similar to the parental virus and more stable [93,94]. In summary several manufacturing processes have been developed to efficiently produce the desired rPVP, each with their advantages and drawbacks.

### 3.2. Antigen Expression on rPVPs

Several processes lead to the expression of foreign antigens on rPVPs. The most commonly used are molecular cloning techniques to fuse sequences coding for the antigen directly within the CP gene construct. In the case of icosahedral viruses such as CMV, CPMV, and AlMV, localization within sequences exposed on the viral surface as well as sites accepting peptide fusions have been well studied [88,105]. In general, insertions of 10–50 amino acids are well tolerated and are structured in closed loops exposed on the surface of the virus. For example, most successful fusions with CPMV were achieved by inserting epitopes within loops between amino acids 22 and 23 of the S protein [43,46,51]. Nevertheless, others have managed to obtain stable particles using N-terminal or C-terminal fusions with AlMV and TBSV, respectively, even if the N-terminal region is known to be important for particle formation for some icosahedral viruses [71,96]. However, in some cases, only 20%–30% of rPVP CPs express the fused antigen [71]. In the case of rod-shaped viruses, *N*-terminal and C-terminal fusions are the most commonly used [56,57,64,74,77]. This allows surface expression without causing destabilization of the structure. In addition, specific and well-defined sites in the CP sequence, other than the C- and N-termini, were also shown to accept fusion without destabilization while allowing surface expression of fused antigens and recognition by the immune system [106,107,108]. However, the tolerated size of peptides fused to rod-shaped viruses is usually more restricted [58,109]. Therefore, in order to fuse bigger peptides or complete proteins, fusion processes other than molecular cloning have been developed. One such technique consists in biotinylating the CP to attach streptavidin-linked proteins or peptides [60,62]. Others also incorporate a reactive lysine in the sequence of the CP to chemically conjugate peptides using a heterobifunctional linker [67,68,100] or perform copper catalyzed azide-alkine cycloaddition to covalently link antigens to the viral capsid [52,110]. Spontaneous conjugation due to electrostatic and/or hydrophobic interactions between foreign antigens and viral CP can also happen and are further stabilized by formaldehyde treatment [101,102]. However, antigen insertion may potentially induce viral particle destabilization [70,109,111,112]. Apart from the size and the localization of the peptide, its charge and isoelectric point can also impact the particles’ capacity to assemble [109,112,113]. In some cases, the generation of mixed particles allows for the formation of stable rPVPs [60]. For example, in order to avoid interference with particle assembly, the insertion of the foot-and-mouth disease virus 2A peptide in the cDNA construct of PVX benefits from a ribosomal skipping process to produce mixed particles made of recombinant and wild-type CPs [58,59]. Finally, the localization of conjugated antigens may also affect the ability to elicit an immune response against fused antigens by influencing the accessibility and conformation of the antigen and thus the immunogenicity of the rPVP [54,110,111]. In summary, with new techniques developed and a better comprehension of factors influencing the stability and immunogenicity of rPVPs, we are better equipped to generate effective vaccines and it is now possible to conjugate very large peptides [59,114] or even complete protein antigens to such particles [62].

### 3.3. rPVPs as Vaccines to Induce Humoral Immune Responses

Most of the currently used vaccines induce a humoral response upon administration, which will then protect individuals against infection or the appearance of disease (reviewed in [115]). As it is the case for current vaccines, plant viruses used as vaccine platforms are also able to trigger the production of antibodies. Not only are these platforms able to induce IgGs, mainly found in serum after sub-cutaneous, intra-peritoneal, and to some extent intra-nasal injections, IgAs are also found in mucosa after intra-nasal and oral administration [44,50,51,53,56]. Plant viruses used as carriers for foreign epitopes can therefore efficiently induce the production of both systemic and mucosal antibodies following administration by various routes, which broadens their potential targets since not all pathogens will require the same antibody response to be cleared from the host. The structure of epitopes presented on the surface of rPVPs will depend on their localization and size. When in an optimal localization, epitopes will adopt a structure similar to their native conformation, allowing antibodies to recognize the virus or bacteria against which they were mounted [54,59]. As such, antibodies from HCV-infected patients were able to recognize a CMV engineered virus expressing the R9 peptide from HCV [70], even though these patients were never exposed to the rPVP. rPVP-based experimental vaccines were also shown to be as effective or even more effective at inducing an antibody response compared to commercially available vaccines [83], peptides alone [61,62,73,74,75], or peptides conjugated to keyhole limpet haemocyanin (KLH) [43,51]. Moreover, these vaccines are often administered in conjunction with less toxic adjuvants, compared to Freund’s Complete Adjuvant (which is not approved for human use), like QS-21 and QuilA (saponin-based) or RIBI [47,48,64], or even without the use of any adjuvant [44,56,57]. Plant viruses also often require less peptide to induce efficient antibody responses compared to commercially available vaccines or peptides conjugated to KLH [41,46].

Immunization in different experimental animal models not only generated specific antibodies but also protected against challenge with various viruses, bacteria, or tumor cell lines when epitopes were presented on CPMV [42,43,45,48,116], PVX [60,61], Bamboo Mosaic Virus (BaMV) [82,83], or TMV [41,63,64,65,66]. Protection against challenge (sometimes lethal) is thought to be partially achieved by neutralizing antibodies, generated following immunization with engineered CPMV [43,47,51,56,85], PVX [56], and Plum Pox Potyvirus [85]. This protection can be broadened to various strains and species of the same pathogen using conserved epitopes, which are usually weakly immunogenic. When such epitopes are presented on the surface of rPVPs, their immunogenicity is increased, allowing for the production of a more effective antibody response and therefore a broader protection of the host [41,49,110]. This has been demonstrated for peptides 10 and 18 of the outer membrane protein F of *Pseudomonas aeruginosa* presented at the surface of CPMV [45,117], as well as for the R9 peptide of HCV presented on PVX [58]. This broad recognition was also observed when mannose was engineered to be displayed on CPMV inducing antibodies interacting with various of its analogues and derivatives [52].

Part of the mechanism by which plant virus carriers induce the production of antibodies is hypothesized to proceed through cross-linking of the BCR [49]. The presentation of many epitopes in close proximity might favor such cross-linking, leading to the proliferation of B lymphocytes, presentation of epitopes to T cells, and differentiation of B lymphocytes into antibody-producing plasma cells [17]. In agreement with this, Nicholas *et al.* demonstrated that higher antigen expression on the surface of CPMV particles provided a better immune response [51]. It also explains why viral platforms seem more efficient at inducing antibodies than peptide-KLH conjugates, for example [43,51].

### 3.4. rPVPs as Vaccines to Induce Cellular Immune Responses

Even though antibodies generated following vaccination are often sufficient to protect against some diseases, elicitation of both cellular and humoral adaptive immune responses is sometimes necessary for protection (reviewed in [115]). The use of plant viruses as epitope carriers has proven effective in inducing cellular immune responses directed towards antigens presented on the particle. We have demonstrated that epitopes fused to the PapMV platform were able to induce the activation of CD8^+^ T cells by a, cross-presentation pathway [33,76,77]. Other rPVPs, such as TMV [40,67,68], CMV [69], CPMV [47] and PVX [57,61] were also shown to generate and enhance CD8^+^ T cell-mediated immune responses against fused epitopes. The main correlate for induction of effective T-cell responses seems to be production of IFN-γ. IFN-γ production was observed when peripheral blood mononuclear cells (PBMCs) from healthy patients or patients infected with HCV were incubated with AlMV expressing a respiratory syncytial virus epitope [72] or CMV expressing the R9 epitope from HCV [69,70], both demonstrating that human PBMCs can be activated by rPVPs, while the latter also demonstrates that the epitope is efficiently processed and presented, leading to the potent activation of CD8^+^ T cells (*i.e.*, IFN-γ production). Cellular immune responses also correlated with protection against challenge with tumor cell lines B16-OVA and Eg.7-OVA [67], Lymphocytic Choriomeningitis Virus [77], and Foot and Mouth Disease Virus [84].

### 3.5. rPVPs Used as Immunomodulators and Adjuvants

In order to generate an effective immune response, APCs must be activated and present antigens to cells of the adaptive immune system [118]. As previously mentioned, rPVPs possess many suitable characteristics for them to be taken up by APCs, processed, and presented to T cells. Indeed, DCs were shown to be activated following TMV [40,67], PVX [56,60], CPMV [55], and PapMV [77,79,80] immunization. Activated DCs upregulate various co-stimulatory molecules such as CD40, CD86, CD80, MHCII, MHCI, and CCR7 [60,79] and also produce pro-inflammatory cytokines such as IL-12, TNF-α, IL-6, and IFN-α [60,78,79,80,81]. DCs are not the only cell population that was shown to be activated following immunization with rPVPs since B lymphocytes, macrophages, and NK cells can also upregulate activation markers following rPVP encounter [55,80].

Since rPVPs can be efficiently taken up by APCs and induce their activation, they can potentially be used as adjuvants to enhance the effectiveness of concomitantly administered vaccines. We have indeed demonstrated that PapMV can be used as an adjuvant in combination with various types of vaccines. When PapMV was administered jointly with bone marrow-derived DCs presenting OVA, cellular immune responses towards OVA were enhanced, leading to better protection against a *Listeria monocytogenes-*OVA challenge in mice [79]. Similar protection was observed when PapMV was administered in mice in concert with the seasonal trivalent influenza vaccine (TIV) [81]. When used as an adjuvant, PapMV was also shown to enhance the production of antibodies directed against TIV [73,80,81]. PapMV has also been shown to be able to prime the immune system and protect mice from an influenza infection on its own, without being fused to an epitope or being administered with a separate vaccine [78]. Finally, it was shown to induce a broader immune response against TIV antigens, providing protection against strains of influenza not contained within the vaccine [81]. To our knowledge, PapMV is the first rPVP used as an adjuvant or an immunomodulator specifically to prime the immune system.

The mechanisms by which rPVPs activate APCs are slowly being uncovered. We have identified one such mechanism in the PapMV system as being the recognition of nucleic acid found within rPVPs that induces APC activation. Single-stranded RNA (ssRNA) within PapMV particles is in fact recognized by TLR7 in endosomes of APCs, mainly plasmacytoid DCs, leading to the production of IFN-α [60,79]. The lack of activation of murine splenocytes upon administration of PapMV monomers further illustrates the importance of ssRNA for its adjuvancy properties [79]. Of note, despite RNA serving as the major immunomodulatory molecule following PapMV administration, the use of RNA alone cannot replace rPVPs since it would be rapidly degraded following administration due to the abundance of host RNAses found within the blood or other bodily fluids. The viral capsid therefore provides protection from degradation to the RNA molecule, allowing it to be efficiently delivered to endosomes. Plant virus particles thus carry their adjuvant properties inside a protective capsid [80], explaining their versatility both as vaccines and adjuvants.

## 4. Conclusions and Perspectives for rPVPs

Although there is still much research to be done before rPVPs are used as vaccines in humans, great achievements have been made in recent years in this field. Several production and antigen expression methods have been developed and improved, thus leading to the generation of many promising candidate vaccines. Beyond demonstrating that rPVPs are effective platforms to generate both humoral and cellular immune responses against fused antigens, we now know that rPVPs are efficiently recognized by the immune system of mammals, which efficiently activates the innate immune system. Therefore, rPVPs possess attractive intrinsic adjuvant properties that can be used for immunomodulatory purposes. This has important implications for future vaccine design and opens the door for new applications. A strong testimony to the recent achievements made in using rPVPs as novel vaccines is the recent entry of two rPVPs in clinical trials, PapMV as an adjuvant for the influenza vaccine [119] and AlMV as a vaccine against malaria [120]. In light of this, it seems very likely that in the near future plant viruses will be used in humans to address unmet medical needs as prophylactic and therapeutic vaccines and immunomodulators against infection or cancer.
